# Development and initial qualitative evaluation of a novel school-based nutrition intervention – COOKKIT (Cooking Kit for Kids)

**DOI:** 10.1186/s12889-023-16598-4

**Published:** 2023-09-07

**Authors:** Simon Pini, William Goodman, Elizabeth Raby, Chris McGinley, Aurora Perez-Cornago, Fiona Johnson, Rebecca J. Beeken

**Affiliations:** 1 Leeds Institute of Health Sciences, University of Leeds, Leeds, UK; 2 Helen Hamlyn Centre for Design, Royal College of Art, London, UK; 3 Nuffield Department of Population Health, Cancer Epidemiology Unit, University of Oxford, Oxford, UK; 4 University College London, London, UK

**Keywords:** School, Cooking, Children, Nutrition, Low-income, Qualitative

## Abstract

**Background:**

Excess weight and an unhealthy diet are risk factors for many cancers, and in high income countries, both are more prevalent among low income families. Dietary interventions targeting primary-school aged children (under 11) can improve healthy eating behaviours, but most are not designed to support the translation of skills learnt in the classroom to the home setting. This paper assessed attitudes and approaches to cooking and eating at home, and the potential to enhance engagement in healthy eating through the COOKKIT intervention.

**Methods:**

COOKKIT is an intervention to deliver weekly cooking classes and supportive materials for low-income families to maintain healthy eating at home. Preliminary qualitative interviews were conducted with teachers and parent–child dyads from a range of primary schools in the UK to explore attitudes, barriers and facilitators for healthy eating and inform the development of COOKKIT. Following implementation, ten children (8–9 y/o) participated in post-intervention focus groups, alongside interviews with teaching staff and parents.

**Results:**

Thematic analysis identified five themes under which to discuss the children’s experience of food, cooking and the impact of COOKKIT: Involving children in planning and buying food for the family; Engaging children in preparing meals at home; Trying to eat healthy meals together in the midst of busy lives; Role-modelling; and Balancing practicalities, information and engagement when delivering cooking classes.

**Conclusions:**

Results suggest COOKKIT provides engaging and easy to follow in-school resources for children and school staff with take-home kits facilitating continued engagement and reinforcing lessons learned in the home environment. Importantly, participants highlighted the combination of healthy eating information, applied practical skills and low costs could support families to continue following the COOKKIT advice beyond the intervention, suggesting further evaluation of COOKKIT is warranted.

**Supplementary Information:**

The online version contains supplementary material available at 10.1186/s12889-023-16598-4.

## Background

Excess weight and an unhealthy diet are risk factors for several cancers, and many other chronic health conditions including type-diabetes, liver disease, heart disease, depression and early mortality [1–6]. Around 6% of UK cancer cases can be attributed to excess weight or dietary factors, including low intake of fruit, vegetables, and fibre, and high consumption of processed meats in relation to colorectal cancer [6]. In the UK there is also a socioeconomic gradient in weight and obesity prevalence in people of all ages, which is particularly strong in children; data from the National Child Measurement Programme shows obesity prevalence is twice as high in primary aged pupils in the most need of economic support compared to those with the least economic needs [7]. Dietary intake is also socially patterned, with children from lower socioeconomic status families eating more processed foods and fewer fruit and vegetables [8].

Obesity is a complex health issue, influenced by a wide range of determinants including the social, physical and economic environment (social determinants) [9]. Childhood obesity rates double during primary school [10], and so supporting children to have healthier diets during this period may be particularly important. One approach to improving children’s diets and preventing excessive weight gain, is to improve nutrition knowledge and teach cooking skills at an early age via school or community-based interventions. Indeed, one of the key recommendations within the World Health Organisation’s report on Ending Childhood Obesity [11], is to *‘Make food preparation classes available to children, their parents and carers,’* and there is some evidence that learning cooking skills early in life is associated with better diet quality and lower obesity rates in adulthood [12]. A 2014 systematic review concluded cooking interventions have the potential to positively influence children’s orientation to food and food related behaviours [13], and several primary school (5-11y/o) based cooking interventions have shown an impact on children’s nutritional knowledge, willingness to try new foods, cooking skills, and engagement in meal preparation [14, 15].

A small number of cooking interventions have specifically targeted young people from priority populations and marginalised backgrounds [16–20]. The majority of these studies have been school-based, with one employing a ‘cooking-camp’ approach [16]. Evaluated using qualitative or mixed methods, these studies have shown that cooking interventions within this population can improve cooking competence and self-efficacy of cooking skills [16, 17, 20]; increase familiarity, acceptance and consumption of vegetables (especially those specifically featured in classes) [16, 17, 20]; and increase involvement in food preparation [16, 17, 20].

However, despite some evidence for the viability of primary school based interventions, there is less research on their translation into the home environment. Barriers to home cooking and healthy eating may be more difficult for families living in low-income homes to address due to limited budgets and time/resources to research and implement solutions. Limited research has been carried out to test this, and the majority of interventions to date have not included elements designed specifically to support the translation of skills learnt in the classroom to the home setting. The COOKKIT (Cooking Kit for Kids) intervention was developed to address the above issues for low-income families through an engaging cooking and nutrition school-based class, which includes “take-home” meal kits and shopping lists designed to transfer and maintain skills and knowledge in the home environment. The aim of this initial qualitative evaluation was to explore attitudes and approaches to cooking and eating at home, and the potential to enhance engagement in healthy eating through the development and initial evaluation of the COOKKIT intervention.

## Methods

### Study design

This study employed a pragmatic epistemology to assess the feasibility and acceptability of delivering a cooking intervention to primary school aged children. Qualitative interviews were carried out with teachers and parent–child dyads to explore attitudes, barriers and facilitators for healthy eating, alongside preferences for a feasible, engaging school-based intervention. Following the delivery of COOKKIT, focus groups were conducted with participating pupils, face-to-face interviews with teaching staff, and telephone interviews with parents to understand experiences of the novel intervention.

### The COOKKIT intervention (see supplementary files for full details and resources)

Initial key features of the COOKKIT intervention were developed by researchers with expertise in nutrition, cancer and behaviour change, who drew on their experience and a review of existing interventions from the academic and grey literature [12, 21–35]. A steering group of teachers, parents and organisations involved in the preparation and delivery of ‘meal kits’ or cookery classes, provided feedback on preliminary design ideas. Findings from pre-intervention qualitative interviews informed the final design for this intervention evaluation, and teachers ensured teaching materials were appropriate and deliverable. Dieticians assessed the dietary content and provided feedback on the recipes, which were also trialled with families prior to the intervention evaluation. A designer was employed to ensure the intervention was child friendly and engaging. The resulting intervention consisted of four weekly cooking classes supported by a manual (Tab. 1) and take home meal kits. The intervention was conducted after school and children were invited to assist in the delivery for peer role-modelling. A stepped approach gradually introduced children to new cooking skills and healthy eating information. Each of the lessons involved 30 min of introductory nutritional information (including risks of future cancer and other illnesses) and cooking skills, followed by 90 min of cooking.

**Table 1:** Overview of COOKKIT intervention

Week	Overview of the cooking classes
1	**Starter:** Children to identify kitchen hazards from a worksheet **Main lesson:** To make a “Rainbow Rumble” and learn key skills of bridge and claw technique for cutting safely
2	**Starter:** Children to play a game to identify the highest fibre food **Main lesson:** To make a “Jewel Couscous”, learn how to safely use the kettle and identify which ingredients will be high in fibre
3	**Starter:** Learn benefits of low calorie food and identify what these would be **Main lesson:** To make a “Veggie Fiesta”, learn how to use the hob safely and how to add flavour without using salt
4	**Starter:** Learn how to get protein in diets without going for red meat **Main Lesson:** To make a “Tuna Pasta Parcel”, learn how to safely use the oven and the benefits of eating fish

**Fig. 1:**
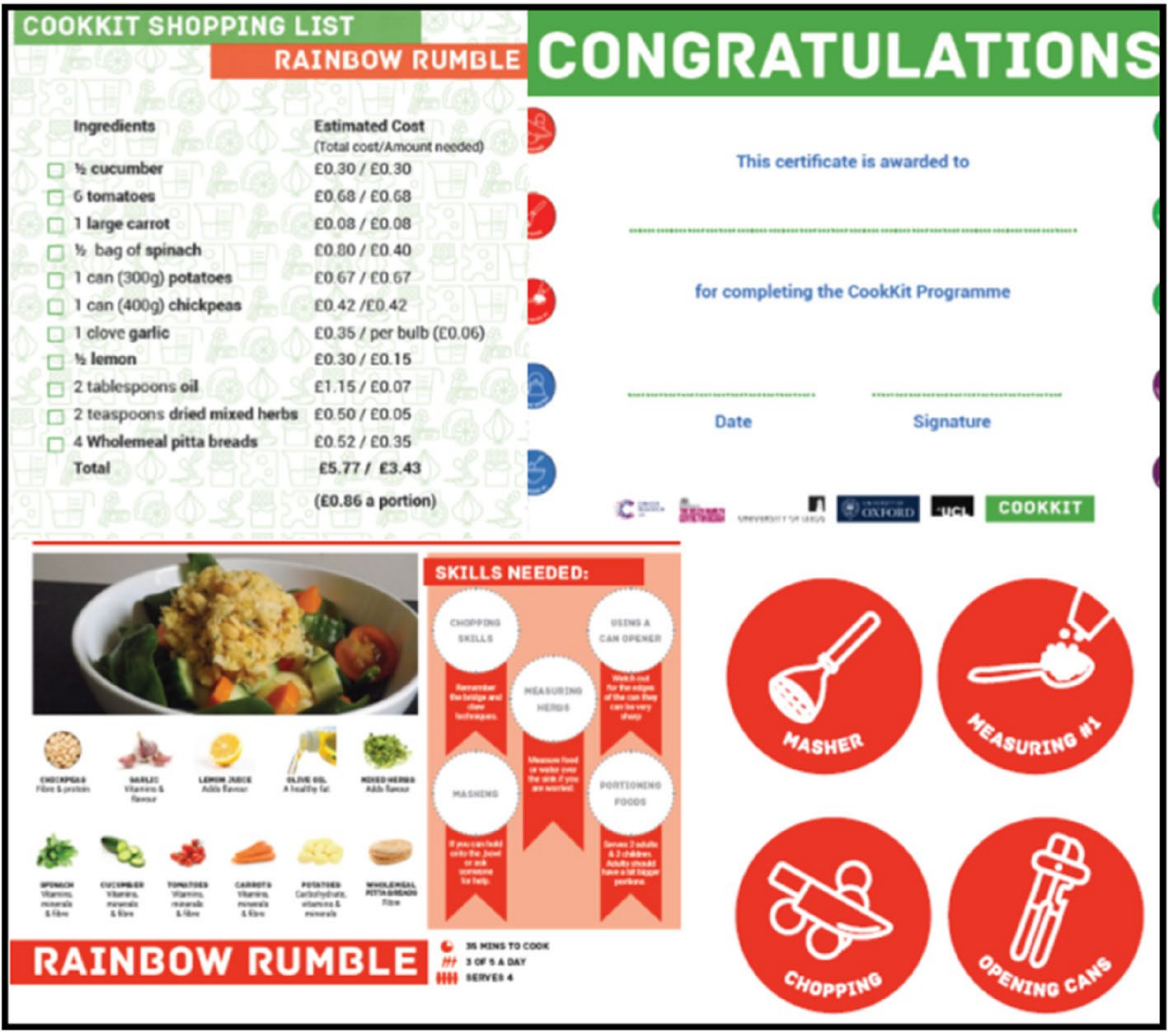
Example contents from COOKKIT take-home packs

The accompanying manual contains teaching materials and lesson plans; recipes with step-by-step guidance for making four different meals; ‘shopping lists’ with nutritional messages; and instructions for preparing “*party bag*” meal kits for the children to take home at the end of each lesson. The party bags contain everything the pupil needs to recreate that week’s meal at home (Fig. 1); all of the ingredients measured out; the recipe; a ‘shopping list’ with ingredient costs to help address financial concerns about healthy eating; a summary of nutritional messages taught within the session; and stickers to reward the pupils for taking part and learning new skills. A certificate was handed out in the final week to the children that completed the intervention.

### Setting and participants

#### Pre-intervention interviews

The pre-intervention interviews were comprised of child/parent dyads and school staff members. These were recruited through social media (Facebook) and participant recruitment websites (Call For Participants). The interviews were conducted at participants’ homes or schools. Children had to be of primary school age (4–11 y/o), as the exact age-group for the intervention was undetermined at this stage. Parents needed to self-identify as a main provider of meals in the family. School staff members were eligible from teaching and non-teaching roles, provided they identified as having experience of cooking education delivery, or knowledge of the food culture and practices of families within their school.

Sample size was based on an estimate of the number required to identify a comprehensive range of themes [36], although in line with the principles of qualitative research this remained flexible to accommodate investigation of novel topics arising during data collection [37, 38].

#### Intervention and post-intervention focus groups and interviews

A school from within a priority population of the UK was recruited to host the intervention through a staff member who participated in the pre-intervention interviews. The school was invited to participate on the basis of a catchment area including neighbourhoods in the most deprived decile of the 2015 index of multiple deprivation [39]. Child participants in the intervention and subsequent focus groups were sampled from two Year 4 (8–9 y/o) classes, reflecting an age at which they were likely to be sufficiently articulate to contribute to the discussion and old enough to be involved in meal preparation and family food decision making. Parents did not participate in the cooking classes, but took part in telephone interviews post-intervention. A member of the school teaching staff led the intervention in a cooking classroom.

The sample size of ten children to participate in the intervention was decided upon in collaboration with the school and the teacher delivering the intervention as to what would constitute a manageable number of children participating in an after-school cookery class. The ten children were divided equally into two focus groups of five.

### Data collection

#### Pre-intervention interviews

The pre-intervention interviews were advertised on social media and participant recruitment websites, with interested participants encouraged to contact the research team for more information and to arrange a time for the interview. Child participants were provided with age-appropriate information and individually assented to participate, alongside the consent of their parent/carer. The children's assent form included questions to check their comprehension of the study, that their participation is voluntary, and that they have the right to withdraw.

Dyad and teacher interviews were semi-structured (supplementary file) and conducted face-to-face with an experienced research assistant. To prepare for the dyad interviews, children were given a study specific questionnaire booklet to self-report their eating habits. This is a form of 'cultural probe' [40], which is a method frequently used in design research where engaging and creative activities are used to provide designers with rich information to inform design concepts. This booklet contained an optional art task to complete (supplementary file), where they were asked to draw their favourite foods, what they buy when they go food shopping and where they eat as a family. Children could then bring the completed booklet to their interview to use as a stimulus for discussion.

These interviews were used to establish current eating practices, attitudes towards food and further refine the intervention.

#### Intervention and post-intervention focus groups and interviews

A collaboration agreement, signed by the head teacher of the primary school, supported the intervention and data collection to take place. Children in two Year 4 classes were sent home with letters and information sheets explaining the cooking classes and parents were asked to send back a signed slip of paper indicating their interest in taking part. As with the pre-intervention interviews, parents provided consent to take part on behalf of themselves and their child, whereas the child provided assent to participate.

Following the intervention, focus groups were conducted with participating pupils, face-to-face interviews with teaching staff, and telephone interviews with parents. These were also semi-structured and conducted by experienced researchers.

### Data analysis

A reflexive thematic analysis was employed as the guiding analytical principle for all data [41, 42], following six stages: 1) familiarisation; 2) coding; 3) searching for themes; 4) reviewing themes; 5) defining and naming themes; 6) writing up. The reflexive approach was selected because of its flexibility to accommodate the variety of participants, time-points and potential directions for the development of the intervention. The first author (SP), not involved in intervention development, conducted all stages of the analysis in consultation with the researcher who conducted the majority of the interviews (WG) and the principle investigator (RB). The pre-intervention interviews were reflected upon by the research team throughout the data collection to assess the coverage of material pertinent to the intervention and make decisions about further recruitment. The formal analysis was conducted following the post-intervention data collection, and rather than considering the participants as separate groups (pupils, teachers and parents), transcripts from all participants were collated as a single analysis group, including data from both pre- and post-intervention time-points. This approach facilitated the generation of overall themes to explain the development of the intervention and participant experiences of cooking and healthy eating behaviours. All participant data contributed to the development of themes, with the focus on the child’s experience of preparing and eating meals. The feasibility and potential benefit of the intervention was assessed in terms of the impact across the identified themes and is detailed in the following sections.

## Results

### Sample

For the pre-intervention interviews there were fourteen parent–child dyads, with ages ranging 6–10 y/o for the children and 30–46 y/o for the parents. Mothers were present and participated in all interviews, and fathers participated in three interviews. Eight members of teaching staff from various primary schools across the UK were interviewed, with ages ranging from 34–60 y/o, seven were female and one male.

A primary school head teacher volunteered to host the intervention. A total of 57 children from two Year 4 classes (8–9 y/o) were approached, and inclusion in the study was decided on a first-come-first-served basis. The final sample consisted of ten children, all of whom participated with a parent. One of the initial ten children withdrew because of other school commitments and was replaced by a child from the “waiting list” of children interested in participating. The sample consisted of six girls and four boys, nine of whom were eight years, and one nine years. Two pupils had dietary requirements; one was vegetarian and the other had coeliac disease, and recipes were adapted to these children’s needs.

### Themes

Analysis of the transcripts identified five themes under which to discuss the children’s experience of food, cooking and the impact of COOKKIT.

- Involving children in planning and buying food for the family
- Engaging children in preparing meals at home
- Trying to eat healthy meals together in the midst of busy lives
- Role-modelling
- Balancing practicalities, information and engagement when delivering cooking classes

Themes are described below with example extracts from participants. Extracts are labelled with a study number and brief description of the participant.

### Involving children in planning and buying food for the family

#### Pre-intervention

We found that children liked to have input into the family meal planning and food shopping, and could be frustrated when their suggestions were denied. However, parents described how involving the children in this process was complicated because it was more time consuming and often resulted in more expensive or wasteful shopping. Managing cost was an important part of food shopping for families, who were conscious that buying food their children would not eat was a waste of the limited resources available to them. Families who reported successfully and regularly involving their children in planning and shopping, often described giving them defined roles, such as finding particular items in the shop, or giving them discreet choices, such as selecting which type of pasta to buy. Regardless of the level of involvement, most families acknowledged their children were more likely to eat the food if they were involved in buying or choosing it.

##### Post-intervention

The shopping lists provided to children following the intervention helped them to be more directly involved in purchasing the "*right kinds of food”*. Alongside actively involving children, the shopping lists encouraged parents to buy healthier ingredients and many reported to be surprised how inexpensive it could be to buy ingredients for healthy home cooking. Similarly, school staff members felt the intervention was relatively inexpensive to provide and gave families positive messages about the cost of healthy eating.

*“My [pupils] were actually talking about feeling fuller and healthier because they were eating healthy, so we had that conversation at one point, which was good. “We can make this and it’s only going to cost 30p a portion,” I think that is a big selling point for a family that are thinking about every penny.” – TS01 - School staff member, post-intervention*

### Confidence, skills and opportunities to prepare meals at home

#### Pre-intervention

The majority of parents described wanting to involve their children in food preparation at home. Prior to the intervention, this was achieved to some extent, but was usually limited to simple baking, or tasks like mixing and stirring. Again a key factor limiting involvement in preparing meals was time, but other important issues were safety and not having child friendly equipment, creating mess, organisation, having other children to care for, and wasting food if mistakes were made.

*“It takes longer because you do have to supervise, you do have to think ‘what can they do?’ and sometimes you just want to get on and do it.” – 106- Parent, pre-intervention*

In the pre-intervention interviews, children regularly suggested wanting to bake cakes and other sweet dishes as part of the cooking classes. Although COOKKIT focussed on healthy savoury meals, rather than baking, the children responded well to the focus on healthy eating and school staff reported being surprised by how successful this was. Having fun and unusual names for the dishes, alongside colourful materials, seemed to be an important component of children’s successful engagement.

*“I liked the names, they were fun and it made us laugh, which is always good. And then they were like, “Cake! Cake! Cake!” and I thought oh we’re in trouble because they want to make some fun baking after school. But actually they were fine about it …they were actually quite happy to make whatever we said, which I thought was very lovely.” – TS01- School staff member, post-intervention*

#### Post-intervention

The most frequently reported perceived benefit of the intervention was how it facilitated children being actively engaged in preparing food at home. Having the ingredients weighed-out and portioned in advance made repeating the meals at home easy to initiate. Parents and children described improvements in cooking skills, especially in relation to skills like chopping and grating, but also safely using kitchen equipment such as hobs and kettles. Several families reported buying the child-friendly equipment used in COOKKIT for use at home, such as the child-friendly knives.

*“I didn’t know you could get child friendly knives…because before I would never let him chop things up or anything. When he was doing it at the cooking club he came home and he was like, “Mum, I can chop you know.” So I let him chop.” – CP04 - Parent, post-intervention*

*“I learnt how not to set the house on fire!” – FG02 - Child, post-intervention*

Children, parents and school staff described seeing an increase in confidence, self-esteem, and enthusiasm because of having improved skills and knowledge about preparing meals, as well as a sense of ownership of the process.

*“I feel more confident of doing it because I always used to think that I messed stuff, but now I have been in cooking club it has given me that more energy and maybe I could make more spices into my curry and stuff like that.” – FG02- Child, post-intervention*

“*I do feel like I’ve watched them flourish in their confidence over the four weeks” – TS01 - School staff member, post-intervention*

This was further enhanced by the easy to follow recipes with accompanying pictures, which reminded the children of the steps they had followed during the COOKKIT lessons.

*“I think she felt great about that because she has brought it back herself and we have been able to cook it and she’s told us what to do because she has done it at school. Yes, it was really, honestly, fantastic, it was…I’ve seen a different side to her, definitely.” – CP01 - Parent, post-intervention*

### Trying to eat healthy meals together in the midst of busy lives

#### Pre-intervention

Despite it being the preferred option of all participants, eating together as a family was complicated by pressures of time, food preferences, parental work, and after school activities. This resulted in many families cooking different meals for different family members at different times, which could be “*chaotic*” and leave little time for planning healthy meals.

*“I think we just never get round to it, everything just feels rushed, constantly rushed…it’s just really working around what each of the kids like and trying to figure out meals that work for everybody.” – 107 - Parent, pre-intervention*

The majority of parents described intending to make healthy choices about what they and their family ate, but not always feeling able to achieve this because of time pressures impacting on planning and preparing healthy meals. Time pressure could sometimes result in turning to ready meals or takeaways, the latter being mainly described as an option ideally only chosen for a treat.

*“[the reason I don’t always cook healthy meals is] I haven’t been organised. That’s the truth. Or we have been out for a long day and I can’t be bothered.” – 101 - Parent, pre-intervention*

Pre-intervention the children demonstrated some awareness of healthy eating, but often their unwillingness to try new foods could be a limiting factor in family meal times. Parents described making occasional attempts to address this at home, with some parents strongly encouraged healthy and diverse eating habits, whereas others tended to adapt to their children’s preferences. School was reported as a key source of healthy eating information through interventions, guidance and rewards. School staff reported observing children making healthy eating decisions when choosing meals at school, but also said that lunches brought with them from home would often have unhealthy content.

*“I think a lot of the choices that they make within school you see…they’ve clearly got that knowledge that actually, yes, they’re thinking oh I’ll have a bit of tuna on my plate and then I’ll have some salad with it rather than just going for the pizza and the beans because it’s easy.” – 208 - School staff member, pre-intervention*

#### Post-intervention

Children, parents and teaching staff all reported the COOKKIT intervention improved children’s understanding of healthy eating choices and encouraged families to try new healthy recipes at home.

*“We learnt about the healthiness of the food because since we have been using vegetables, it helps you get that better sleep and it helps you be more healthier and it helps with a lot of other things.” – FG02 - Child, post-intervention*

All families reported the children had recreated COOKKIT recipes at home, which provided impetus for all family members to engage in communal meal times, shared meal preparation and trying new foods together.

### Role-modelling

#### Pre-intervention

Through their interviews, children revealed influences on their eating behaviours and attitudes to food. Role-modelling seemed to be an important factor, with children picking up on cues from the people and environment around them. Dads were often cited by children as a source of fun when shopping for food, but also as the parent who displayed less healthy food behaviours themselves and used sweet treats as rewards or incentives.

*“Daddy doesn't like baked beans and I don't like baked beans…Daddy has biscuits every single day of the week!” – 107 - Child, pre-intervention*

*“I like it when daddy makes my pack lunch because he doesn't know the rules.” – 107 - Child, pre-intervention*

School staff appeared very conscious of their role in modelling healthy eating behaviours through the food they were seen to be eating in school and the rewards they were offering pupils. Alongside demonstrating healthy food behaviours themselves, school staff often tried to recognise and acknowledge instances of pupils demonstrating healthy eating and promote conversations with/between pupils about this subject.

*“We try and eat healthily in front of the children and…we are not encouraged to bring sweets anymore if it’s birthdays but raisins and things like that” – 207 - School staff member, pre-intervention*

The influence of other children was present for several participants who positively described their experiences of baking or cooking at birthday parties, or learning from watching older siblings in their family. This was not always positive for encouraging healthy food behaviours, but appeared to have a significant impact on the children’s motivation to follow the behaviours they observed. Conversely, two children described how much they enjoyed helping their own younger siblings.

#### Post-intervention

COOKKIT utilised the idea of peer role-modelling by encouraging children to take on different roles in the lessons and show each other how to complete the tasks. Children were keen to help and act as role models during the lesson.

*“Lots of kids asking me, “Can I help? Can I help?” So I like that peer leadership, I think that’s something we could work with again.” – TS01 - School staff member, post-intervention*

Several parents said they saw this role-modelling transferring to home, with children leading on preparing the meals and showing younger siblings, and often parents, how to follow the recipes. Parents also described the motivation their children got from seeing their peers all engaging in the same tasks and enjoying working together.

*“I think it helped that he has seen his friends doing it as well. I think that really helped to give him that bit of confidence. Because he does look up to his friends” – CP03 - Parent, post-intervention*

### Balancing practicalities, information and engagement when delivering cooking classes

#### Pre-intervention

Prior to the intervention, several children described having already enjoyed and engaged in cooking classes at school or in the local community. However, parents described frustration with some of these classes, which were in high demand, meaning places were difficult to secure, they happened at irregular intervals or at inconvenient times, could be “*boring”* or “*repetitive*”, and could be expensive to join and recreate at home.

School staff members described difficulties with providing regular cooking classes in their schools because of cost, staffing, time and the priority placed on other areas of the curriculum.

*“I just think that the curriculum now is so focused, there is so much to cram in because schools are under pressure to achieve those academic scores, the data is so important, that teachers are reluctant to do those practical activities on a regular basis” – 202 - School staff member, pre-intervention*

#### Post-intervention

COOKKIT provided a template for cooking classes, which was enjoyed by pupils and school staff, and appreciated by parents. School staff found the templates for delivering the classes very effective and easy to follow. Some made useful adaptations throughout the weeks, such as giving each pupil a specific role, or sometimes a *“micro-role”* (e.g. chopping an onion or garlic) to make sure they were all focussed and had a specific task that was their own. They also encouraged pupils to prepare and pack their own party-bags to take home, which was an efficient use of time, as well as connecting the pupils to what they were taking home.

Prior to the intervention, the majority of parents and pupils suggested a cooking class they could participate in together would be their preference. However, there was also feedback that after-school or evening classes would be more complicated to attend because of work commitments and responsibility for other children. COOKKIT addressed these issues by offering classes aimed at the children, but with resources and encouragement to be able to subsequently involve parents and family at home.

*“I think it would be easier from our point of view for it [the focus of the intervention] to be the children. And then hopefully the children then educate at home. I think it would impact on less kids if we were having families in.” – 206 - School staff member, pre-intervention*

As well as practical concerns, school staff reflected during the intervention that it was preferable to have time with just the pupils, so they could develop and reinforce cooking skills and health information.

*“I do think it’s quite nice to see the families cook together, but I started to think you do need a longer period where we are supervising the kids on our own and then we can get the health messages over at the same time as having a good time.” – TS01 -School staff member, post-intervention*

Alongside enjoyable and engaging lessons, COOKKIT provided impactful health information and applied cooking and eating behaviours that were reported to translate into the home environment. Teaching staff reported accounts of the lasting impact of the intervention, for example:

*“They can’t remember all of it but they understood that it was about having a balanced diet and being healthy. They understood that brown pasta is more healthy than white. They might not know why, but they understand the messages, the rules, and that’s helpful because it’s a bit of knowledge when you’re at the shops, isn’t it, that could make a difference.”- TS01- School staff member, post-intervention*

The recipes offered as part of COOKKIT were well received by children, which was appreciated by parents who had suggested their children needed encouragement to try healthy food and to make it as appealing as less healthy alternatives.

*“It’s almost like teaching children that healthy food is delicious…I worry maybe at [other cooking classes] they tried too hard to make it really healthy, that’s when the kids don't like it.” – 107 - Parent, post-intervention*

All participants reported the materials provided during and following the intervention were simple to understand, even when used at home without the classroom support. School staff also described this as offering good differentiation in the lessons.

*“The kids this morning, when I was talking to them, were talking about understanding the pictures even if they can’t read the recipe” – TS01 - School staff member, post-intervention*

*“You made them fun, so we were in the mood to make them because you put smiley faces on them….And plus when you put it in a bowl, you have showed the bowl, how it looks like.” – FG03 - Child, post-intervention*

All participants described wanting the intervention to continue and be available across other year groups.

*“It’s fun…In year 5 can we still do it?” – FG01 - Child, post-intervention*

*“I was quite disappointed that was going to be our last one. She enjoyed it. She came out here and spoke about it all the time and she really enjoyed cooking at home as well.” – CP01 - Parent, post-intervention*

## Discussion

For children, school and home are key environments in which to address excess weight and poor diet as long term risk factors for many cancers and other non-communicable diseases. The COOKKIT intervention was developed with the aim to provide knowledge and resources to children in school to facilitate improvements in cooking skills and health eating behaviours at home. This evaluation showed that the COOKKIT intervention was a viable and engaging method for schools to adopt to try and improve the healthy eating behaviours and knowledge of pupils from priority populations. Previous literature has shown that healthy lifestyle programmes [14] and “hands-on” involvement in cooking and cooking skills [15] can increase young people’s fruit and vegetable intake and confidence in preparing healthy meals. In-keeping with this, our qualitative data suggest 8-9y/o children, and their parents, were able to understand and engage with nutritional information and healthy eating decision making. This engagement was facilitated through step-by-step instructions, fun and interactive age-appropriate materials, and applied practical involvement through defined roles.

After school education programmes [17], engagement with diverse foods [18], and parent and child cooking classes [19], have all shown potential of transferring cooking skills and healthy eating knowledge from the classroom to the home environment. Importantly, COOKKIT directly addressed this knowledge transfer through novel “take-home” materials aligned with the lessons, which provided a clear and tangible way of recreating and repeating learning from one environment to the other. Although other school-based cooking interventions have offered take-home recipe sheets [43, 44], few have included pre-portioned ingredients which may facilitate first-time preparation of the recipe in the home-setting. A US based study that aimed to promote family cooking by distributing take home food kits with ingredients and recipes following in-school food preparation and tasting activities has previously demonstrated the potential benefits of this approach for increasing liking of vegetables [18]. As well as the take-home recipes, and pre-portioned ingredients framed as ‘party bags’, we also provided shopping lists with indicative pricing to further support engagement from the children’s families and to encourage future communal meal preparation and eating.

As with previous studies employing cooking classes and instruction [14–20], children’s cooking skills, associated confidence and self-efficacy appeared to improve following COOKKIT. During COOKKIT the peer role-modelling element of the experience, both providing and observing, became an important addition within the classroom lessons. Parents reported this modelling of behaviour transferred to the home environment through the children’s confidence around demonstrating their new skills and knowledge to their own younger siblings and parents. Role-modelling appeared to be a key factor in the way children created and reinforced their view of eating behaviours, but also provided an area that could be specifically targeted by the intervention as a way of engaging and influencing children’s perceptions and behaviours around healthy eating.

For COOKKIT to be a viable intervention for schools to provide, but also for the messages to have longevity within families, healthy eating interventions need to be cost effective. Considering the target demographic, this was a key focus of the COOKKIT intervention. School staff were encouraged by the affordability of the intervention and the ability to tailor the content and structure to their particular school and local population. Previous research has shown that low-income families make judgements about food cost in terms of raw monetary cost, but also in relation to “unappreciated costs” such as food waste, how long food will last and whether it will satiate the family [45]. Providing the families with pre-portioned ingredients initially and then shopping-lists for future purchasing, helped to emphasise the potential for healthy eating at affordable and sustainable prices, and was welcomed by parents.

### Limitations

Whilst this study has a strong grounding in the experiences of young people and families, the findings and conclusions drawn from this initial development and evaluation are limited to the experiences of the small sample of young people, families and school staff recruited to the study. Additionally, socio-economic status was not collected for the pre-intervention participants, so the diversity in the foundations of the intervention is not known.

The participating school for the intervention were engaged in the study and able to find time to accommodate the study. This may not represent all schools in priority areas, who could experience additional challenges with capacity and resourcing that would need assessing on an individual basis.

This initial pilot study was not able to assess the extent to which the benefits of COOKKIT were sustainable over time. Sustained and measurable benefits for low-income families will be essential to understand the long term viability of applying COOKKIT more widely.

## Conclusion

The COOKKIT intervention provides a feasible approach to address this issue, and now requires further testing in larger and more diverse populations, including investigation of the longitudinal impact on the dietary knowledge, cooking skills and eating behaviours of both children and their families. Improving healthy eating, especially amongst children from priority populations, has the potential to improve dietary intake, reduce obesity, and ultimately lower risk factors for cancer and other non-communicable diseases.

### Supplementary Information

**Additional file 1. **

## Data Availability

The datasets generated and/or analysed during the current study are not publicly available as they are primarily transcripts from interviews and focus groups with children. However, there is potential for some anonymised data sharing upon reasonable request to be sent to Dr. Simon Pini s.pini@leeds.ac.uk.

## Abbreviation

COOKKIT: Cooking Kit for Kids
